# Tailoring Electro/Optical Properties of Transparent Boron-Doped Carbon Nanowalls Grown on Quartz

**DOI:** 10.3390/ma12030547

**Published:** 2019-02-12

**Authors:** Mattia Pierpaoli, Mateusz Ficek, Michał Rycewicz, Mirosław Sawczak, Jakub Karczewski, Maria Letizia Ruello, Robert Bogdanowicz

**Affiliations:** 1 Department of Materials, Environmental Sciences and Urban Planning, Università Politecnica delle Marche, 60131 Ancona, Italy; m.l.ruello@univpm.it; 2 Department of Metrology and Optoelectronics, Faculty of Electronics, Telecommunication and Informatics, Gdańsk University of Technology, 80-233 Gdańsk, Poland; matficek@pg.edu.pl (M.F.); micrycew@student.pg.edu.pl (M.R.); 3 Polish Academy of Sciences, The Szewalski Institute of Fluid-Flow Machinery Fiszera 14, 80-231 Gdansk, Poland; miroslaw.sawczak@imp.gda.pl; 4 Faculty of Applied Physics and Mathematics, Department of Solid State Physics, Gdansk University of Technology, 11/12 Narutowicza Str., 80-233 Gdansk, Poland; jakub.karczewski@pg.edu.pl

**Keywords:** carbon nanowalls, boron-doping, chemical vapor deposition, band-gap, transparent electrode

## Abstract

Carbon nanowalls (CNWs) have attracted much attention for numerous applications in electrical devices because of their peculiar structural characteristics. However, it is possible to set synthesis parameters to vary the electrical and optical properties of such CNWs. In this paper, we demonstrate the direct growth of highly transparent boron-doped nanowalls (B-CNWs) on optical grade fused quartz. The effect of growth temperature and boron doping on the behavior of boron-doped carbon nanowalls grown on quartz was studied in particular. Temperature and boron inclusion doping level allow for direct tuning of CNW morphology. It is possible to operate with both parameters to obtain a transparent and conductive film; however, boron doping is a preferred factor to maintain the transparency in the visible region, while a higher growth temperature is more effective to improve conductance. Light transmittance and electrical conductivity are mainly influenced by growth temperature and then by boron doping. Tailoring B-CNWs has important implications for potential applications of such electrically conductive transparent electrodes designed for energy conversion and storage devices.

## 1. Introduction

Most commercial transparent electrodes are usually made of indium tin oxide (ITO) due to its good electrical performance (~10 Ω/square), while the limited amount of indium resources raises concerns in the European Union as well as globally. For this reason, the scientific community has moved to seek new solutions [1,2]; among those, carbon-based transparent conducting materials are extensively applied nowadays in many optoelectronic [3,4], biosensing [5,6], energy storage, and conversion devices [7,8,9]. Carbon nanostructures are particularly attractive due to their unique three-dimensional structure with a high surface area and specific surface interactions [10]. Alternative nanomaterials, such as carbon nanowalls (CNWs), have held the attention of the scientific community due to their structural characteristics in mono-, bi- and tri-dimensional arrangements [11,12,13], including excellent electrical and thermal conductivity. Their unique geometrical shape and morphology cause CNWs to possibly be an interesting material for field emitters [14]. Field emitters based on CNWs are expected to be free from Joule heating, in contrast to structures based on carbon nanotubes [15]. Good cooperation with gold nanoparticles has resulted in the development of a new type of Surface Enhanced Raman Scattering (SERS) substrate based on CNWs which is known for its high sensitivity [16]. The additional use of H-termination makes CNWs hydrophobic and very stable in a humid environment [17]. Zhang et al. [18] have shown that vertically aligned graphene can deliver superior thermal management capabilities, which has been revealed by scalable frost removal. Moreover, that effect was achieved on glass substrates, creating potentials for future transparent ’green-warmth’ devices.

Nevertheless, standard chemical vapor deposition (CVD) fabrication of CNWs usually requires a relatively high temperature of up to 1000 °C of growth or catalyst on the substrates (e.g., Cu or Ni) to obtain effective films [11]. This usually results in optically opaque films which are useless in the field of optical application. The solution is the utilization of plasma-enhanced processes at low temperatures, along with growth time limitation to minimize the absorbance of CNW layers [11,19,20,21]. The uniform and direct growth of few-layer graphene on a CuNi grid on the float glass has been demonstrated at the grown temperature of 500 °C by Chen et al. [21]. Furthermore, carbon nanowalls are wall-like “graphitic” sheets [22] that can also be fabricated by catalyst-free direct growth methods using fused silica as a substrate by radio frequency (plasma enhanced chemical vapor deposition (PECVD)). Nong et al. [22] have revealed that CNWs post-decorated with CdTe can work as a high-performance photo-anode with a current density of 3 mA cm^−2^.

Recently, authors have reported a novel way to achieve PECVD growth of enhanced carbon nanowalls on silicon wafers by in-situ boron doping, which has attracted interest due to a tunable band gap, high conductivity, high mechanical robustness and, unluckily, high optical absorbance [5,6,23]. For this reason, together with boron-doped nanocrystalline diamond thin films, their growth over a transparent substrate, such as quartz, is of current interest.

Growing doped-diamond or carbon nanowall films at low temperatures (less than 500 °C) is a main concern because many substrate materials, such as borosilicate glass, are unstable at higher temperatures, which are generally required for standard CVD diamond growth. However, the temperature affects the morphology of the nanomaterial. For this reason, Remes et al. investigated how the addition of oxygen to the gas mixture in a CVD setup allows for the growing of nanodiamond film at relatively low temperatures, which are, however, not conductive because the oxygen in plasma prevents the incorporation of boron into the diamond lattice [24].

A change in the conductivity of almost ten orders of magnitude was found by Gajewski et al. for a change in the boron concentration by five orders of magnitude, and in this study the different conduction mechanisms predominating at different boron doping levels were discussed [25]. 

In this paper, we demonstrate the direct growth of boron-doped nanowalls on optical grade fused quartz. Optical grade fused quartz is commonly used as a transparent and insulating amorphous oxide, with high surface hydrophilicity and low thermal conductivity in various optical and electronics devices. Merging glass with CNWs enables the fabrication of rigid transparent conductive electrodes for a number of applications. The growth of boron-doped carbon nanowalls (B-CNWs) with different doping concentrations (0, 1.2, 2, 5, and 7.5 part per thousand of [B]/[C] ratio) and different heating temperatures (400 °C, 550 °C, 700 °C, and 850 °C) was investigated in particular. The influence of temperature growth and boron on the structure of the obtained material, surface morphology, molecular composition, and electrical and optical properties are the focus of this paper. To the best of our knowledge, the effects of B-CNW growth on optical grade fused quartz for optical applications have not yet been reported.

## 2. Materials and Methods

### 2.1. B-CNWs Growth Method

Prior to CVD growth, fused quartz substrates (Continental Trade Sp.z.o.o., Warszawa, Poland) were pre-treated using the microwave PECVD (MWPECVD) system (SEKI Technotron AX5400S, Tokyo, Japan) for 20 min with H_2_ at 500 °C and 1000 W microwave power. This hydrogenation pre-treatment was supposed to change surface termination and wetting properties, which improve diamond seeding efficiency. Before the process, the substrates were seeded by spin-coating them in a diamond slurry [6]. The base pressure inside the chamber was 10^−4^ Torr. Several B-CNW films were fabricated using the following process conditions: gas mixtures H_2_, CH_4_, B_2_H_6_, and N_2_ with a total flow of 328 sccm; process pressure of 50 Torr; microwave power up to 1300 W; and microwave radiation of 2.45 GHz. For boron-doped samples, diborane (B_2_H_6_) was used at different levels (0, 1.2, 2, 5, and 7.5 part per thousand of [B]/[C] ratio) as an acceptor precursor. During the process, the substrate holder was heated up to the desired temperature by an induction heater (400 °C, 550 °C, 700 °C, and 850 °C) which was controlled by a thermocouple. Initially, three samples were grown for 7.5, 15, and 30 min (at 700 °C and 2000 ppm [B]/[C]) in order to choose the best process duration in terms of light transmittance and electrical conductivity. A 15 min process was chosen in order to meet both needs.

All the sample characteristics are summarized in Table 1.

### 2.2. Surface Morphology

Scanning electron microscopy via a FEI Quanta FEG 250 scanning electron microscope (SEM, FEI, Hillsboro, OR, USA), using a 15 kV beam accelerating voltage with a secondary electron–Everhart-Thornley (SE-ETD) detector working in high vacuum mode (pressure 10^−4^ Pa) was used to observe the structure of the B-CNW surfaces. The program used for data visualization and analysis was Gwyddion (v.2.40, Czech Metrology Institute, Brno, Czech Republic).

### 2.3. Raman Spectroscopy

The molecular composition of the deposited films was studied by means of Raman spectroscopy using a Raman microscope (InVia, Renishaw, UK). Spectra were recorded over the range 200–3500 cm^−1^ with an integration time of 5 s (10 averages) using an argon ion laser emitting at 514 nm and operating at 5% of its total power (50 mW). Data were smoothed (using a Savitzky-Golay method: 15 points, second polynomial order) and the baseline (approximated with a cubic polynomial) subtracted and normalized.

### 2.4. Spectroscopy Ellipsometry

Spectroscopic ellipsometry analysis was conducted with a Jobin-Yvon UVISEL phase-modulated ellipsometer (HORIBA Jobin-Yvon Inc., Edison, Rosemead, CA, USA). The investigated wavelength region was between 250 and 800 nm. The experiments were carried out at room temperature using an angle of incidence fixed at 60° and a compensator set at 45°. DeltaPsi software (v. 2.4.3, HORIBA Jobin-Yvon Inc., Edison, Rosemead, CA, USA) was employed to determine the spectral distributions of refractive index n (λ) and extinction coefficient k (λ) of the B-CNW films.

### 2.5. Electrical Conductivity

Electrical conductivities were measured at room temperature by a four-point probe placed in a straight line with equal spacing (s = 1.5 mm). Needle-like probes with a radius of 100 μm were utilized. The size of the samples was 1 × 1 cm^2^. A correction factor of 0.86 was considered during conductivity estimation, due to the finite sample size, and the layer thickness was estimated by spectroscopic ellipsometry measurements. The current was gradually increased from 0 up to 300 μA, with a step of 10 μA, with a source meter, and the voltage on the internal probes was measured with a Keithley 2400.

### 2.6. UV Visible Spectroscopy

UV-visible spectroscopy was measured using a double beam spectrophotometer (UV-9000 Metash, Shanghai, China) for the 200–1000 nm range with a scan step of 1 nm and a scan filter of 10. In this spectrophotometer, a deuterium lamp and a tungsten halogen lamp are used as light sources, a double beam optical system with 1200 lines/mm grating is implemented, and silicon photodiodes play the role of detectors. The wavelength repeatability is about 0.2 nm and the wavelength accuracy is equal to ±0.3 nm. Photometric accuracy is equal to about ±0.3 %T, and photometric repeatability is equal to 0.2 %T, where %T is percent transmittance.

In order to evaluate the band gap energy (E_g_) of the samples, (αhν)^n^ were plotted against the photon energy (hν), according to the well-known relation [26]

(1) $${\left(\alpha h\nu \right)}^{n}=A\left(h\nu -E_{g}\right),$$

where A is a constant, hν is the photon energy, and n depends on the type of allowed transition in the material. It has a value equal to 1/2, 2, and 3/2 for directly allowed transitions, indirectly allowed transitions, and directly forbidden transitions, respectively.

While both allowed direct and indirect transitions were computed, those of a direct type are proposed in this study.

## 3. Results

**Figure 1:**
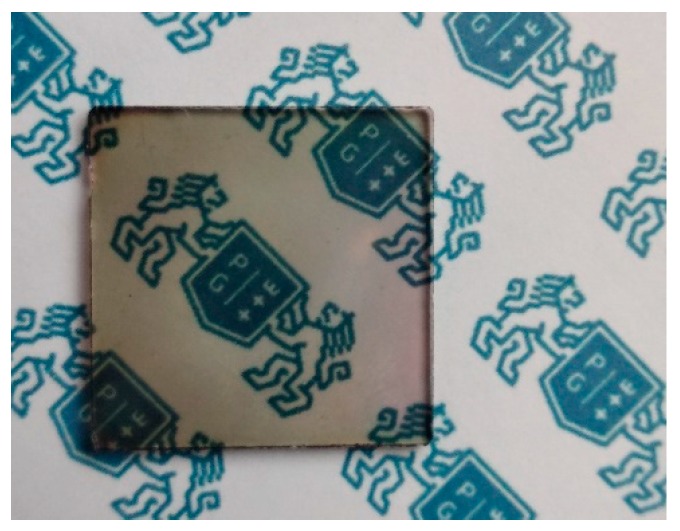
Picture of the fabricated sample: growth at 700 °C with a 2000 ppm [B]/[C] ratio.

The achieved transparency of B-CNW growth on quartz is shown in Figure 1, which shows a picture of the sample growth at 700 °C with a 2000 ppm [B]/[C] ratio.

### 3.1. Investigation of the Growth Process

**Figure 2:**
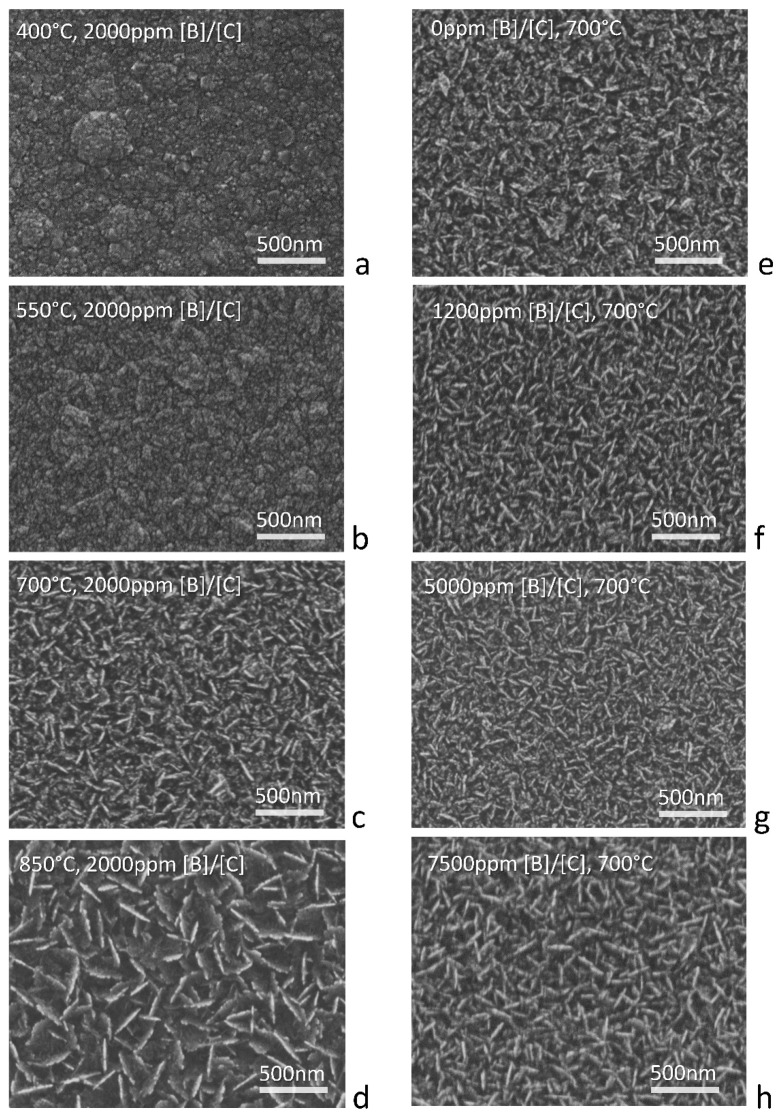
Scanning electron microscopy (SEM) picture of B-CNW growth, respectively, at (**a**) 400 °C, (**b**) 550 °C, (**c**) 700 °C, and (**d**) 850 °C at 2000 ppm [B]/[C] ratio and with (**e**) 0, (**f**) 1200, (**g**) 5000, and (**h**) 7500 ppm [B]/[C] at 700 °C. Sample (**c**) belongs to both series.

Morphology of boron-doped carbon nanowalls plays an important role in the light transmission process and also affects the conductivity of the deposited material. Figure 2a–d shows the SEM images of B-CNW growth, respectively, at 400 °C, 550 °C, 700 °C, and 850 °C at a 2000 ppm [B]/[C] ratio and for Figure 2c,e–h at 0, 1200, 2000, 5000, and 7500 ppm [B]/[C] at 700 °C. The size of the B-CNWs is 1 cm × 1 cm.

It was found that a randomly vertically aligned wall structure belonged to the substrate only for samples with a temperature of process higher than 700 °C. B-CNWs are homogeneously distributed all over the substrate and they have sharp top edges.

For B-CNWs with a temperature of process of 400 or 550 °C the structure resembles nanoislands—one of the growth stages suggested by Hiramatsu et al. [27].

**Figure 3:**
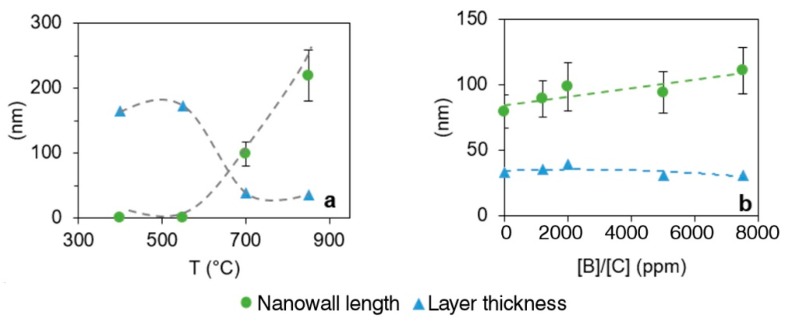
(**a**) Nanowall length and layer thickness for B-CNW growth at 400 °C, 550 °C, 700 °C, and 850 °C at a 2000 ppm [B]/[C] ratio; (**b**) nanowall length and layer thickness for B-CNW growth at 0, 1200, 2000, 5000, and 7500 ppm [B]/[C] at 700 °C.

In our studies, a significant effect of temperature on B-CNW length was observed (Figure 3a). Samples with a temperature of process of 850 °C contain nanowalls approximately two times longer than for 700 °C samples. The distance between longer B-CNWs is greater than between the shorter ones. Temperature causes a decrease in the thickness of B-CNWs, which is correlated with the length of the nanowalls.

Boron incorporation, in different ratios at the same growth temperature, into the B-CNWs lattice causes a slight decrease in the thickness of B-CNWs (Figure 3b), which is in good agreement with research suggested by Sobaszek et al. [6].

It is worth pointing out that wall length is slightly positively influenced by the boron level in the gas phase. The sample 5000 ppm [B]/[C] at 700 °C has approximately 20 nm longer walls than the sample 0 ppm [B]/[C] at the same temperature of process.

### 3.2. Absorbance Spectrum

**Figure 4:**
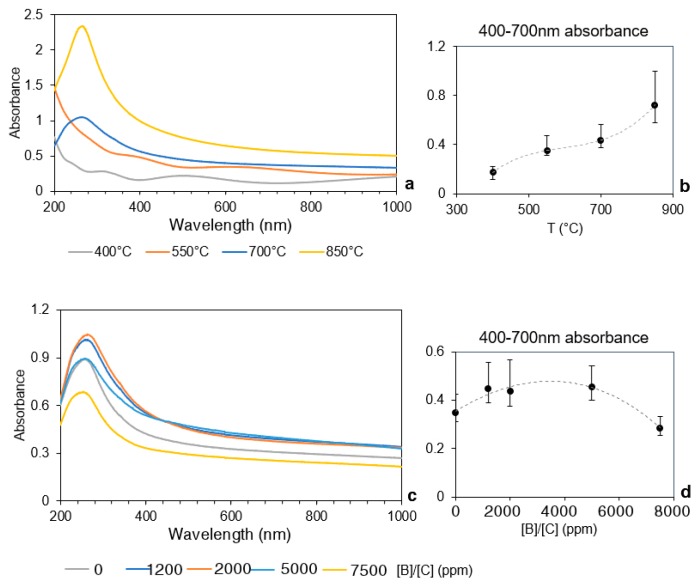
(**a**) UV-visible absorbance spectrum and (**b**) 400–700 nm average absorbance for B-CNW growth at 400 °C, 550 °C, 700 °C, and 850 °C at 2000 ppm [B]/[C] ratio; (**c**) UV-visible absorbance spectrum and (**d**) 400–700 nm average absorbance for B-CNWs at 0, 1200, 2000, 5000, and 7500 ppm [B]/[C] at 700 °C.

Absorbance spectra for sample growth at different temperatures are shown in Figure 4a. 

**Figure 5:**
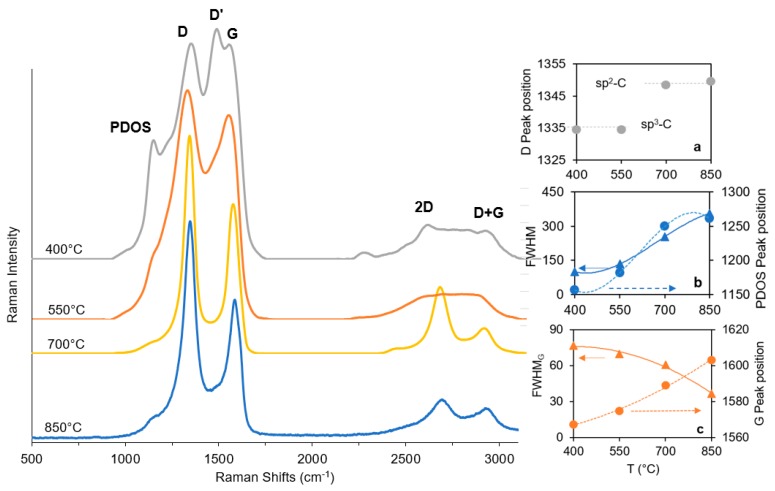
Raman spectra of sample growth at 400 °C, 550 °C, 700 °C, and 850 °C at a 2000 ppm [B]/[C] ratio. In the inserts (**a**–**c**), peak position and full-width at half-maximum (FWHM) are reported.

The absorbance for wavelengths of between 400 nm and 700 nm ranges between 0.2 and 0.8 with increasing growth temperature. In particular, a prominent change in average absorbance is observed in the 200 nm to 400 nm wavelength region (Figure 4b). This is due to a change in the morphology and composition of the B-CNW layer, as confirmed by SEM (Figure 2) and Raman (Figure 5). As the temperature rises, we see a more distinct shape of the walls. Also as the temperature rises, we get an absorption peak at the wavelength of 200–350 nm. The absorption for 850 °C for 270 nm is 2.4, which corresponds to a transmission below 0.002%. This spectroscopic feature consistently observed in the UV range is known to originate from the peak in the joint density of states for a simple graphene layer [28] which occurs at the M point of the Brillouin zone at E = 2γ_0_ and is usually known as the plasmon resonance. For carbon nanotubes, this feature becomes shifted and split due to the more complex band structure, as has been predicted by several authors [29,30].

The absorbance spectrum for the samples with different [B]/[C] ratios are similar (Figure 4c), while the trend of visible light absorption for samples initially increases and then decreases with a higher [B]/[C] ratio higher than 5000 ppm (Figure 4d).

### 3.3. Raman Spectroscopy

Raman spectra of B-CNWs were analyzed and are reported in Figure 5. In all samples, two main bands at ∼1580 cm^−1^ and ∼1350 cm^−1^ are found, which are generally attributed to the G and D bands, respectively. In particular, Ferrari and Robertson have suggested that the G and D peaks are due to sp^2^ only [31].

The band centered near 1350 cm^−1^ corresponds to the breathing mode of sp^2^ atoms in a ring (D band), and Figure 5a and its intensity correlates with the amount of disorder of sp^2^-hybridized atoms in graphite-like materials. The different samples morphology, previously described and reported in Figure 2, is also highlighted by the presence of the D peak at 1350 cm^−1^ in the Raman spectra for the B-CNW growth at a higher temperature, which corresponds to distortion of the sp^2^ crystal structure; by contrast, for the first two samples (at 400 °C and 550 °C), a peak appears earlier, at 1334 cm^−1^, which can be attributed, instead, to the diamond structure [32].

The wide band in the range 1500–1600 cm^−1^ is dominated by overlapping G and D’ bands.

The G band centered at 1580 cm^−1^ is associated with bond stretching of sp^2^ atoms in rings and chains (G band, Figure 5b). The G peak position is shifted to a higher frequency with higher temperatures and the full-width at half-maximum (G-FWHW) is also found to decrease with temperature. Marchon et al. have reported a similar trend and have linked it to a decrease in sp^3^ content [33]. 

The D’ band observed near 1500 cm^−1^ is usually caused by defects generated via double-resonance processes. The origin of the D’ band in the plasma processed graphite nanomaterials can be associated with nitrogen defects incorporated into the graphene lattice [34,35]. The intensity of the D’ band changes with plasma process temperature. The maximal intensity is observed for B-CNW film synthesized at 400 °C and the band disappears for films deposited at temperatures above 700 °C. Increasing the process temperature results in a reduction of defect concentration, which is also reflected in the narrowing of the D band.

The band at 1220 cm^−1^ is reported to be caused by the effect of boron on the diamond lattice [36]. However, this peak also appears in boron-free CNWs, which makes another hypothesis reasonable. Chen et al. [37] merged their results with theoretical analysis and experimental results from others to identify two additional Raman peaks at around 1168 cm^−1^ and 1271 cm^−1^, in tetrahedral amorphous carbon, linked to the sp^3^ bonding. In our results, reported in Figure 5c, it is possible to observe a distinct peak, ahead of the D band, which redshifts with increasing temperature. This peak can be assigned to the sp^3^ carbons or disordered diamonds and is related to the phonon density of states (PDOS) band [38,39]. Moreover, from the Raman spectra it may be observed that when the boron concentration was increased further from a 2000 ppm [B]/[C] the G band peak upshifted by 6 cm^−1^. A similar result was noted by McGuire et al. [40] for boron-doped single-walled carbon nanotubes.

### 3.4. Optical and Electrical Properties

**Figure 6:**
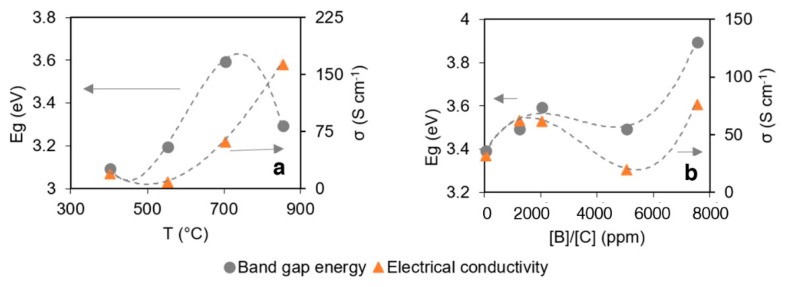
(**a**) Optical bandgap and electrical conductivity for B-CNW growth at 400 °C, 550 °C, 700 °C, and 850 °C at a 2000 ppm [B]/[C] ratio; (**b**) optical bandgap and electrical conductivity for B-CNW growth at 0, 2000, 5000, and 7500 ppm [B]/[C] at 700 °C.

In Figure 6a it is possible to observe how higher electrical conductivity belongs to sample growth at a higher temperature, in a similar fashion to nanowall length, as this parameter particularly affects charge mobility. Longer wall structure delivers higher conductivity, mostly due to the minimized effect of carrier scattering at the inter-wall regions and surface defects. Next, the optical bandgap shows a maximum for the sample growth at 700 °C, which is much larger than the values reported by Kawai et al. [41]. This effect is unrevealed, though it could be attributed to the specific molecular structure caused by boron doping. Boron incorporation into the carbon lattice slightly positively affects nanowall length, which is not directly translated into an enhancement of the electrical conductivity; however, the latter, jointly with the optical bandgap, depends on the [B]/[C] ratio (Figure 6b). Overall, the incorporation of boron into the CNW lattice structure induces the unique effect of enhanced electrochemical performance and improved charge transfer [42,43].

The refractive index of B-CNWs, evaluated at 550 nm, ranges between 2.10 and 2.86 for sample growth at 700 °C, respectively, at 0 ppm and 5000 ppm boron doping levels; these results are comparable to those found by others [44,45]. Temperature also affects the refractive index; however, the variation is smaller and it falls within the previously reported range. The extinction coefficient shows a similar trend, reaching its minimum, equal to 0.83, with the sample growth at 700 °C and 0 ppm and 7500 ppm boron doping levels, and its maximum, equal to 1.24, with the 2000 ppm sample. For sample growth at a temperature of 300 °C and 450 °C, which does not exhibit a nanowall structure, the extinction coefficient is much lower, indicating that the structure is similar to that of diamond-like carbon (DLC) film [46].

## 4. Discussion

Process duration affects mainly the thickness of the nanowall layer. Longer times lead to thicker layers, together with longer and more electrically conductive nanowalls, while light transmittance is negatively affected. For these reasons, the right thickness is the starting point for obtaining a conductive and transparent composite. 

Increasing nanowall length and layer thickness in accordance with boron doping are in contradiction to that reported by Takashi et al. [47] but in agreement with others [31,35,48].

Raman spectra and SEM pictures show that growing B-CNWs in low temperatures brings more defects than with elevated temperatures, with difficulties in developing a well-developed nanowall network on the substrate for temperatures lower than 700 °C, as well as a thinner B-CNW layer. Nanowall length and electrical conductivity are favorable with elevated growth temperatures.

The drop in growth rate versus stage temperature is caused by different surface pyrolysis reaction pathways involving H, CHx, HCN, and CN radicals [6]. The higher temperature favors bonding of CHx radicals towards the formation of a nanocrystalline diamond sp^3^ phase observed in the Raman spectra. The growth rate of the diamond phase is much smaller than the nanowall formation kinetics [6]. 

**Figure 7:**
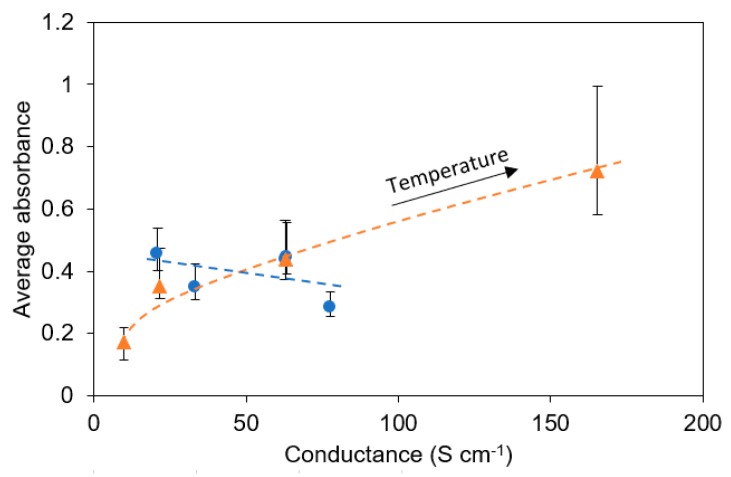
Dependency of the average absorbance on the conductivity of B-CNWs deposited on fused quartz.

The dependency of B-CNW average absorbance in the 400–700 nm range, as a function of the conductance, is reported in Figure 7. It is interesting to observe how the conductance and absorbance are slightly negatively correlated for different boron concentrations (blue ●), while they are positively correlated for different growth temperatures (orange △). 

## 5. Conclusions

In this study, a successful deposition of B-CNWs on a quartz glass substrate was conducted and characterization was carried out in order to study two parameters which strongly affect B-CNW growth and morphology: growth temperature and boron doping.

Process duration affects not only the thickness of the nanowall layer but also the development of the nanowall structure. 

Raman spectrum and SEM pictures show that growing B-CNW in low temperatures brings more defects than with elevated temperatures, with difficulties in developing a well-developed nanowall network on the substrate observed for temperatures lower than 700 °C. Nanowall length and electrical conductivity are favorable with elevated growth temperatures.

Boron incorporation into the carbon lattice slightly positively affects nanowall length, which is not explicitly translated into an enhancement of the electrical conductivity.

These results suggest that it is possible to operate with both parameters to obtain a transparent and conductive film; however, boron doping is a preferred solution to maintain the transparency in the visible region, while a higher growth temperature is more effective to improve conductance.

## Figures and Tables

**Table 1:** Parameters of growth of boron-doped carbon nanowall (B-CNW) films.

Sample ID	Growth Time (min)	Boron Doping [B]/[C] (Part Per Thousand)	Furnace Temperature (°C)
2k_700	15	2	700
0k_700	15	0	700
1.2k_700	15	1.2	700
5k_700	15	5	700
7.5k_700	15	7.5	700
	15	2	400
	15	2	550
	15	2	850

## References

[1] Lu H., Ren X., Ouyang D., Choy W.C.H. (2018). Emerging Novel Metal Electrodes for Photovoltaic Applications. Small.

[2] Huang Q., Zhu Y. (2019). Printing Conductive Nanomaterials for Flexible and Stretchable Electronics: A Review of Materials, Processes, and Applications. Adv. Mater. Technol..

[3] Jo G., Choe M., Cho C.-Y., Kim J.H., Park W., Lee S., Hong W.-K., Kim T.-W., Park S.-J., Hong B.H. (2010). Large-scale patterned multi-layer graphene films as transparent conducting electrodes for GaN light-emitting diodes. Nanotechnology.

[4] Wang Z., Mao S., Baba K., Ito T., Ogata H. (2014). Microwave plasma-assisted regeneration of carbon nanosheets with bi- and trilayer of graphene and their application to photovoltaic cells. Carbon.

[5] Hosu I.S., Sobaszek M., Ficek M., Bogdanowicz R., Drobecq H., Boussekey L., Barras A., Melnyk O., Boukherroub R., Coffinier Y. (2017). Carbon nanowalls: A new versatile graphene based interface for the laser desorption/ionization-mass spectrometry detection of small compounds in real samples. Nanoscale.

[6] Sobaszek M., Siuzdak K., Ryl J., Sawczak M., Gupta S., Carrizosa S.B., Ficek M., Dec B., Darowicki K., Bogdanowicz R. (2017). Diamond Phase (sp3-C) Rich Boron-Doped Carbon Nanowalls (sp2-C): Physicochemical and Electrochemical Properties. J. Phys. Chem. C.

[7] Hassan S., Suzuki M., Mori S., El-Moneim A.A. (2014). MnO_2_/carbon nanowalls composite electrode for supercapacitor application. J. Power Sources.

[8] Lin G., Zhou Y., Wang Y., Yan X., Wu B., Huang F., Fu J., Cheng Q., Yun D. (2017). Direct growth of graphene nanowalls on quartz substrates as transparent conductive electrodes for perovskite solar cells. Funct. Mater. Lett..

[9] Lee K.-T., Park D., Baac H., Han S., Lee K.-T., Park D.H., Baac H.W., Han S. (2018). Graphene- and Carbon-Nanotube-Based Transparent Electrodes for Semitransparent Solar Cells. Materials.

[10] Manawi Y.M., Ihsanullah, Samara A., Al-Ansari T., Atieh M.A. (2018). A Review of Carbon Nanomaterials’ Synthesis via the Chemical Vapor Deposition (CVD) Method. Materials.

[11] Davami K., Shaygan M., Kheirabi N., Zhao J., Kovalenko D.A., Rümmeli M.H., Opitz J., Cuniberti G., Lee J.-S., Meyyappan M. (2014). Synthesis and characterization of carbon nanowalls on different substrates by radio frequency plasma enhanced chemical vapor deposition. Carbon.

[12] Santhosh N., Filipič G., Tatarova E., Baranov O., Kondo H., Sekine M., Hori M., Ostrikov K., Cvelbar U., Santhosh N.M. (2018). Oriented Carbon Nanostructures by Plasma Processing: Recent Advances and Future Challenges. Micromachines.

[13] Al-Jumaili A., Alancherry S., Bazaka K., Jacob M., Al-Jumaili A., Alancherry S., Bazaka K., Jacob M.V. (2017). Review on the Antimicrobial Properties of Carbon Nanostructures. Materials.

[14] Wu Y., Yang B., Zong B., Sun H., Shen Z., Feng Y. (2004). Carbon nanowalls and related materials. J. Mater. Chem..

[15] Teii K., Nakashima M. (2010). Synthesis and field emission properties of nanocrystalline diamond/carbon nanowall composite films. Appl. Phys. Lett..

[16] Dyakonov P., Mironovich K., Svyakhovskiy S., Voloshina O., Dagesyan S., Panchishin A., Suetin N., Bagratashvili V., Timashev P., Shirshin E. (2017). Carbon nanowalls as a platform for biological SERS studies. Sci. Rep..

[17] Krivchenko V.A., Evlashin S.A., Mironovich K.V., Verbitskiy N.I., Nefedov A., Wöll C., Kozmenkova A.Y., Suetin N.V., Svyakhovskiy S.E., Vyalikh D.V. (2013). Carbon nanowalls: The next step for physical manifestation of the black body coating. Sci. Rep..

[18] Zhang N., Li J., Liu Z., Yang S., Xu A., Chen D., Guo Q., Wang G. (2018). Direct synthesis of vertical graphene nanowalls on glass substrate for thermal management. Mater. Res. Express.

[19] Pierpaoli M., Lewkowicz A., Ficek M., Ruello M.L., Bogdanowicz R. (2018). Preparation and characterization of TiO2/carbon nanowall composite on a transparent substrate. Photonics Lett. Pol..

[20] Zhao R., Ahktar M., Alruqi A., Dharmasena R., Jasinski J.B., Thantirige R.M., Sumanasekera G.U. (2017). Electrical transport properties of graphene nanowalls grown at low temperature using plasma enhanced chemical vapor deposition. Mater. Res. Express.

[21] Chen Y.-Z., Medina H., Tsai H.-W., Wang Y.-C., Yen Y.-T., Manikandan A., Chueh Y.-L. (2015). Low Temperature Growth of Graphene on Glass by Carbon-Enclosed Chemical Vapor Deposition Process and Its Application as Transparent Electrode. Chem. Mater..

[22] Nong J., Wei W., Song X., Tang L., Yang J., Sun T., Yu L., Luo W., Li C., Wei D. (2017). Direct growth of graphene nanowalls on silica for high-performance photo-electrochemical anode. Surf. Coat. Technol..

[23] Sankaran K.J., Ficek M., Kunuku S., Panda K., Yeh C.-J., Park J.Y., Sawczak M., Michałowski P.P., Leou K.-C., Bogdanowicz R. (2018). Self-organized multi-layered graphene–boron-doped diamond hybrid nanowalls for high-performance electron emission devices. Nanoscale.

[24] Remes Z., Avigal Y., Kalish R., Uzan-Saguy C., Chack A., Nesládek M. (2004). Structural, optical and electrical properties of nanodiamond films deposited by HFCVD on borosilicate glass, fused silica and silicon at low temperature. Phys. Status Solidi.

[25] Gajewski W., Achatz P., Williams O.A., Haenen K., Bustarret E., Stutzmann M., Garrido J.A. (2009). Electronic and optical properties of boron-doped nanocrystalline diamond films. Phys. Rev. B.

[26] Tauc J. (1968). Optical properties and electronic structure of amorphous Ge and Si. Mater. Res. Bull..

[27] Hiramatsu M., Hori M. (2006). Fabrication of Carbon Nanowalls Using Novel Plasma Processing. Jpn. J. Appl. Phys..

[28] Pedersen T.G. (2003). Variational approach to excitons in carbon nanotubes. Phys. Rev. B.

[29] Dmitrović S., Vuković T., Nikolić B., Damnjanović M., Milošević I. (2008). Plasmon excitations of single-wall carbon nanotubes. Phys. Rev. B.

[30] Takagi Y., Okada S. (2009). Theoretical calculation for the ultraviolet optical properties of single-walled carbon nanotubes. Phys. Rev. B.

[31] Ferrari A.C., Robertson J., Ferrari O., Robertson J.O.H.N. (2004). Raman spectroscopy of amorphous, nanostructured, diamond-like carbon, and nanodiamond. Philos. Trans. A Math. Phys. Eng. Sci..

[32] Haubner R., Moritz R. Raman characterisation of diamond coatings using different laser wavelengths. Proceedings of the Nineteenth European Conference on Chemical Vapor Deposition.

[33] Marchon B., Gui J., Grannen K., Rauch G.C. (1997). Photoluminescence and ramana Spectroscopy in hydrogenated carbon films. IEEE Trans. Magn..

[34] Ye D., Wu S.-Q., Yu Y., Liu L., Lu X.-P., Wu Y. (2014). Patterned graphene functionalization via mask-free scanning of micro-plasma jet under ambient condition. Appl. Phys. Lett..

[35] Bokobza L., Bruneel J.-L., Couzi M., Thakur V.K. (2015). Raman Spectra of Carbon-Based Materials (from Graphite to Carbon Black) and of Some Silicone Composites. C.

[36] Krivchenko V.A., Lopaev D.V., Minakov P.V., Pirogov V.G., Rakhimov A.T., Suetin N.V. (2007). Study of polycrystalline boron-doped diamond films by Raman spectroscopy and optical absorption spectroscopy. Tech. Phys..

[37] Chen Z.Y., Zhao J.P., Yano T., Ooie T., Yoneda M., Sakakibara J. (2000). Observation of sp3 bonding in tetrahedral amorphous carbon using visible Raman spectroscopy. J. Appl. Phys..

[38] Windl W., Pavone P., Karch K., Schütt O., Strauch D., Giannozzi P., Baroni S. (1993). Second-order Raman spectra of diamond from ab initio phonon calculations. Phys. Rev. B.

[39] Roy D., Barber Z.H., Clyne T.W. (2002). Ag nanoparticle induced surface enhanced Raman spectroscopy of chemical vapor deposition diamond thin films prepared by hot filament chemical vapor deposition. J. Appl. Phys..

[40] McGuire K., Gothard N., Gai P., Dresselhaus M., Sumanasekera G., Rao A. (2005). Synthesis and Raman characterization of boron-doped single-walled carbon nanotubes. Carbon.

[41] Kawai S., Kondo S., Takeuchi W., Kondo H., Hiramatsu M., Hori M. (2010). Optical properties of evolutionary grown layers of carbon nanowalls analyzed by spectroscopic ellipsometry. Jpn. J. Appl. Phys..

[42] Siuzdak K., Ficek M., Sobaszek M., Ryl J., Gnyba M., Niedziałkowski P., Malinowska N., Karczewski J., Bogdanowicz R. (2017). Boron-Enhanced Growth of Micron-Scale Carbon-Based Nanowalls: A Route toward High Rates of Electrochemical Biosensing. ACS Appl. Mater. Interfaces.

[43] Fudala-Ksiazek S., Sobaszek M., Luczkiewicz A., Pieczynska A., Ofiarska A., Fiszka-Borzyszkowska A., Sawczak M., Ficek M., Bogdanowicz R., Siedlecka E.M. (2018). Influence of the boron doping level on the electrochemical oxidation of raw landfill leachates: Advanced pre-treatment prior to the biological nitrogen removal. Chem. Eng. J..

[44] Gharibyan A., Hayrapetyan D., Panosyan Z., Yengibaryan Y. (2011). Preparation of wide range refractive index diamond-like carbon films by means of plasma-enhanced chemical vapor deposition. Appl. Opt..

[45] Ficek M., Sobaszek M., Gnyba M., Ryl J., Gołuński Ł., Smietana M., Jasiński J., Caban P., Bogdanowicz R. (2016). Optical and electrical properties of boron doped diamond thin conductive films deposited on fused silica glass substrates. Appl. Surf. Sci..

[46] Robertson J. (2002). Diamond-like amorphous carbon. Mater. Sci. Eng. Rep..

[47] Itoh T., Nakanishi Y., Ito T., Vetushka A., Ledinský M., Fejfar A., Kočka J., Nonomura S. (2012). Electrical properties of carbon nanowall films. J. Non-Cryst. Solids.

[48] Kurita S., Yoshimura A., Kawamoto H., Uchida T., Kojima K., Tachibana M., Molina-Morales P., Nakai H. (2005). Raman spectra of carbon nanowalls grown by plasma-enhanced chemical vapor deposition. J. Appl. Phys..

